# Comparison of suicide rates in the USA and Australia between 1921 and 2020: major shifts in youth and elderly suicide rates over a century

**DOI:** 10.1192/bjo.2025.10863

**Published:** 2025-10-30

**Authors:** Sydney Z. Ma, Shahid Ullah, Stephen Allison, Stephen R. Kisely, Jeffrey C. L. Looi, Tarun Bastiampillai

**Affiliations:** Department of Psychiatry, Northern Sydney Central Coast Local Health District, St Leonards, Australia; College of Medicine and Public Health, Flinders University, Adelaide, Australia; Consortium of Australian-Academic Psychiatrists for Independent Policy and Research Analysis (CAPIPRA), Canberra, Australia; School of Medicine, Princess Alexandra Hospital Southside Clinical Unit, Greater Brisbane Clinical School, Medical School, The Faculty of Health, Medicine and Behavioural Sciences, The University of Queensland, Woolloongabba, Australia; Social Psychiatry and Epidemiology Research Unit, School of Medicine and Psychology, The Australian National University, Canberra, Australia; Department of Psychiatry, Monash University, Clayton, Australia

**Keywords:** Durkheim, suicide trends, youth suicide, elderly suicide, gender

## Abstract

**Background:**

Trends in the US and Australian suicide mortality have shifted over the last 100 years, with notable differences between age groups and genders.

**Aims:**

This study compared overall and gender- and age-specific suicide rates from 1921 to 2020 in the USA and Australia to determine long-term variation for each country.

**Method:**

Suicide data (1921–2020, inclusive) were obtained from the US Centers for Disease Control and Prevention and the Australian Institute of Health and Welfare. Poisson regression was used to assess whether suicide rates between groups were significantly different.

**Results:**

Overall suicide rates were higher in the USA compared to Australia, from 1921 to the 1940s, but were similar from the 1950s onwards. While male suicide rates fluctuated, female suicide rates were relatively stable (except for Australian women in the 1960s). In the USA and Australia, suicide rates for young males have significantly increased since the 1950s, while they have decreased for the older male population since the 1940s.

**Conclusions:**

While overall national suicide rates were relatively stable over 100 years apart from during war and economic depression, male suicide rates in the USA and Australia experienced significant age-related changes over the century. These include major declines in males aged over 65 years but also an increase in suicides for those aged between 15 and 44. Suicide rates across age groups have therefore converged, regressing towards the mean for all age groups combined.

Suicide is one of the leading causes of death in both the USA and Australia.^1,2^ The major rise in male youth suicide rates in the USA and Australia has been a particular public health concern. Despite national suicide prevention programmes and improved population access to first-line treatments such as antidepressants and psychotherapy in these countries, overall national suicide rates have not fallen below the 100-year means.^3^

Of course, not all suicides are related to mental illness, and Durkheim noted the sociocultural factors that may also determine suicide rates, such as social alienation or anomie.^4^ Furthermore, Durkheim proposed that each country has a more or less stable secular rate of suicide, depending on national sociocultural factors, unless there are major events such as war or economic depression, or increased access to lethal means such as firearms.^4^ Given comparable experiences of economic depression and war in the USA and Australia over the last century, overall patterns of suicide have been broadly similar.

For instance, suicide rates in the USA increased during the Great Depression, then fell during World War II (WWII).^5^ The second half of the 20th century saw relative stability of US suicide rates, maintained until the millennium.^5,6^ However, during the 21st century, US suicide rates started to increase again, partially because of opioid misuse.^7^ During the last 100 years in Australia, suicide rates experienced similar fluctuations related to the Great Depression (increase) and WWII (decrease).^3^ In both countries, there was a barbiturate-related suicide rate increase in the 1960s, particularly in Australia.^8–10^ Males had higher suicide rates, with greater variation, compared to women, in both countries.^8,9,11^

Previous research has found that Australian suicide rates were trending upwards towards the overall and gender-specific 100-year means in the early 21st century.^3^ However, these trends may disguise significant changes in age-specific suicide rates. We extend research on age-specific suicide rates and Durkheim’s prediction by comparing and contrasting Australia and the USA, which is also a former British colony with a displaced First Nations population, where diversity increased with immigration in the late 20th century. Both are predominantly English speaking, and Australian popular culture is heavily influence by that of the USA. However, there are also significant cultural differences, such as more pervasive gun culture in the USA with higher rates of firearm ownership.^12^

Australia and the USA have both experienced a major rise in youth male suicide rates, beginning in the second half of the 20th century, and a concurrent major reduction in elderly male suicide rates.^7,8,13^ However, we have been unable to locate direct statistical comparison of US and Australian suicide rates with gender and age stratification for the century between 1921 and 2020. Continuous suicide data are available for these countries, so we compared the 100-year suicide rates in the USA and Australia.

## Method

### US data

US suicide rates were obtained from the Centers for Disease Control and Prevention (CDC).^1^ Annual suicide rates by year of registration of death, age and gender were obtained using ICD codes for years 1921–2020 inclusive.^(1)^ Mortality data from 1999 to 2020 were obtained from CDC Wide-ranging Online Data for Epidemiologic Research (CDC WONDER) using ICD-10 codes X60–X84 and Y87.0. ICD-9 and ICD-8 codes E950–E959 were used to obtain data for 1979–1998 and 1968–1977, respectively. Data for 1921–1967 inclusive were obtained from CDC unpublished workbooks that can be accessed via their National Vital Statistics System. Annual age-specific death rates for all ages were calculated by taking the number of deaths over total population per annum that were provided by CDC upon our specific request.

US data were used to compare with Australian data because the USA also had a complete data-set over 100 years (with gender and age stratifications), and we were unable to find a comparable database for other predominantly English-speaking countries for 100 years inclusive of age subdivisions.

### Australian data

Deaths by suicide for Australia were obtained from the Australian Institute of Health and Welfare (AIHW) from their National Mortality Database.^2^ Annual suicide numbers by year of registration of death, age and gender for 1921–2020, inclusive, were coded under ICD-10 codes X60–X84 and Y87.0 by the Australian Bureau of Statistics (ABS). The deaths were provided in 5-year age groups by the AIHW. Annual age-specific death rates for 15–24, 25–44, 45–64 and 65+ age groups were calculated by taking the number of deaths over the total population per annum provided by the AIHW General Record of Incidence of Mortality workbook (GRIM book).^14^

### Statistical methods

Suicide rates were calculated as the number of suicides per 100 000 population. Annual age- and gender-specific and total rates are provided in the data figures for years 1921–2020 inclusive. The age groups were 15–24, 25–44, 45–64 and 65+. A sub-analysis of 10-year age groups for the 65+ population was also performed, which included age groups 65–74, 75–84 and 85+. Ages under 15 were excluded from the study because of extremely low number of suicides in this group.

The suicide incidence rate ratios (IRRs) were calculated by the ratio of the number of deaths by the population at risk for each group in the USA and Australia. The baseline period was chosen as 1921–1930 to compare the rest of the data with the beginning of the study period. The baseline period for the 85+ age group was chosen as 1941–1950 because of the absence of suicide data for this age group before 1941. A Poisson regression model was applied to examine the significant changes of suicide rates between groups. The estimates were calculated using the likelihood ratio method and were expressed as IRRs from the Poisson model. A two-sided test was performed for all analyses, 95% confidence intervals were reported and the level of significance was set at *p* < 0.05. R version 4.2.1 for Windows (R Foundation for Statistical Computing, Vienna, Austria; https://cran.r-project.org/bin/windows/base/old/4.2.1/) was used to analyse the data. See Supplementary Tables available at https://doi.org/10.1192/bjo.2025.10863 for the IRR comparisons.

### Research ethics

No ethical approval or consent was required as this paper does not involve research with humans or animals and relates to de-identified data. The data used in this paper were publicly available from the AIHW and CDC.

## Results

### Trend in suicide rates by gender

**Fig. 1:**
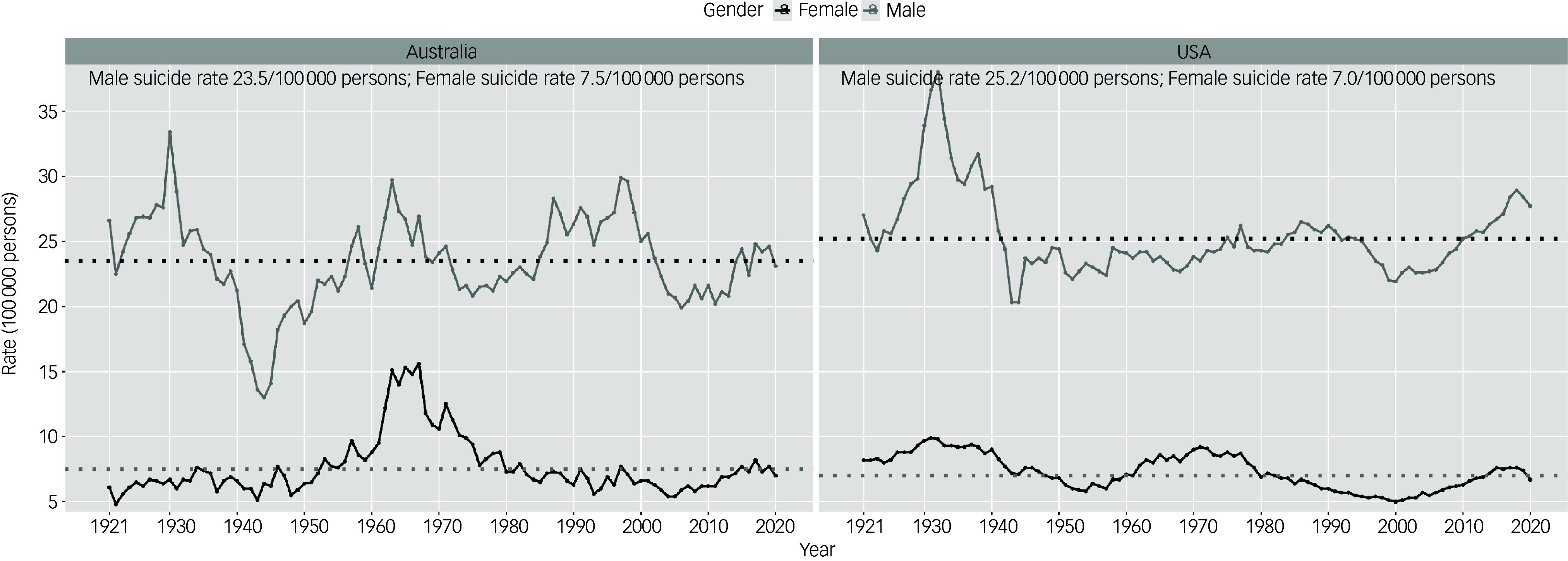
Total suicide rates in the USA and Australia by gender, 1921–2020. The dotted line represents the suicide rates of males and females in Australia and the USA.

From 1921 to 2020, male suicide rates in both the USA and Australia showed marked fluctuations (Fig. 1). The highest rates were observed in the early 1930s (38 per 100 000 in 1932 in the USA, 33.4 per 100 000 in 1930 in Australia). While both countries experienced marked falls in rates from the mid-1940s, especially in Australia where rates in 1944 were 13 per 100 000, the subsequent patterns differed markedly. In the USA, rates remained relatively stable from 1945 until the mid-1990s. By contrast, rates in Australia showed greater volatility from the lowest rate in 1944 (13 per 100 000) through to the 2000s. This included peaks in the 1960s (29.7 per 100 000 in 1963), 1980s (28.3 per 100 000 in 1987) and 1990s (29.9 per 100 000 in 1997). Subsequently, both countries experienced increases in suicide rates in 21st century before they declined from the mid-2010s (recent peak rates are 28.9 per 100 000 in 2018 in the USA, 24.8 per 100 000 in 2017 in Australia).

By contrast, female suicide rates were relatively stable over time, typically ranging between 5 and 10 per 100 000 in both countries (Fig. 1). Notable exceptions include peaks in suicide rates in US women in the 1930s (9.9 per 100 000 in 1931) and 1970s (9.2 per 100 000 in 1971) and a suicide peak among Australian women in the 1960s (15.6 per 100 000 in 1967). Recent suicide rates declined for women from 2015 (7.6 per 100 000) in the USA and from 2017 (8.2 per 100 000) in Australia to 2020.

### Trend in suicide rates by age and gender

**Fig. 2:**
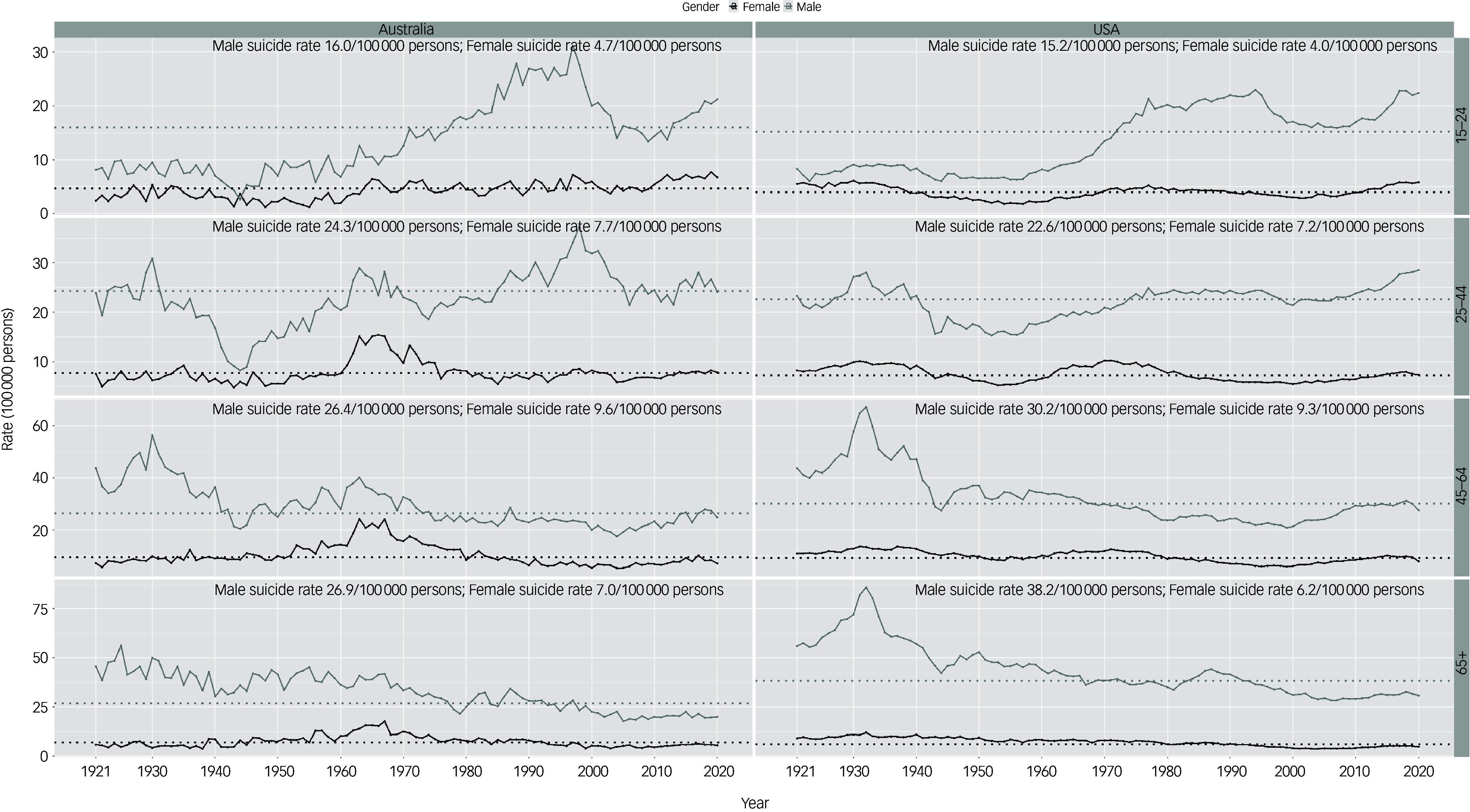
Suicide rates by age groups and gender in the USA and Australia for age groups 15–24, 25–44, 45–64 and 65+, 1921–2020. The dotted line represents the suicide rates of males and females in Australia and the USA.

As shown in Fig. 2, suicide rates were relatively stable for males aged 15–24 in both the USA and Australia until the 1960s, other than a trough in 1944 (2.6 per 100 000) in Australia, followed by major increases extending through to the 1990s. US male suicide rates in this age group peaked at 23 per 100 000 in 1994, while they peaked at 31.2 per 100 000 in 1997 in Australia. Troughs in suicides occurred in the 2000s for both countries relative to neighbouring years (15.9 per 100 000 in 2007 in the USA, 13.4 per 100 000 in 2009 in Australia), followed by recent increases in suicide rates until 2020.

Women aged 15–24 in USA had a relatively stable suicide rate from 1921 to 2020, while the rate experienced a gradual increase in Australia during this time. The US suicide trend displayed two troughs in 1950s (1.8 per 100 000 in 1957) and 2000s (3 per 100 000 in 2003), while the Australian 15–24 female suicide trend was more volatile in terms of year-to-year changes with relatively smaller troughs in the 1950s (1.2 per 100 000 in 1955) and 2000s (3.7 per 100 000 in 2003).

For men aged 25–44, Australia had a more volatile pattern in suicide rate trends when compared to the USA. There was a peak in the early 1930s in both the USA (28 per 100 000 in 1932) and Australia (30.9 per 100 000 in 1930). The lowest male suicide rate for the USA was in 1952 (15.3 per 100 000), while in Australia it was in 1944 (8.2 per 100 000). The rates in the USA showed relative stability between the 1970s and 2010s, while Australia had peaks in the 1960s (28.9 per 100 000 in 1963), 1990s (37.4 per 100 000 in 1998) and 2010s (30 per 100 000 in 2017). The highest suicide rate in Australian 25–44 males was in 1998 (37.4 per 100 000), while the the highest suicide rate in the USA was in 2020 (29 per 100 000).

For women aged 25–44 in the USA, overall data showed a mostly stable pattern other than troughs in the 1950s (5.2 per 100 000 in 1953) and 2000s (5.4 per 100 000 in 2000). In Australia, suicide rates in women aged 25–44 were relatively stable other than a unimodal peak in the 1960s (highest rate in 1966 of 15.4 per 100 000).

There were peaks in suicide rates in men aged 45–64 in 1932 (67.2 per 100 000) in the USA and 1930 (56.3 per 100 000) in Australia. In the USA, there was a trough in 45–64-year-old male suicides in the late 1990s (20.9 per 100 000 in 1999), while in Australia there was a peak in the 1960s (40.1 per 100 000 in 1963) and a relatively minor trough compared to neighbouring years in the 2000s (17.6 per 100 000 in 2004).

US women aged 45–64 experienced relatively stable suicide rates between 1921 and 2020, with minor troughs compared to neighbouring years in the 1950s (8.3 per 100 000 in 1954) and 1990s (6 per 100 000 in 1999). Australian women aged 45–64 displayed a unimodal peak in suicides in the 1960s (24.2 per 100 000 in 1963), with relatively stable rates otherwise.

Men older than 65 in the USA showed a peak in suicide rates in the 1930s (85.7 per 100 000 in 1932), while the rate gradually declined from 1950 to 2020 with a minor peak in the 1980s (44.2 per 100 000 in 1987). In Australia, there was a gradual decrease in the 65+ male suicide rate from 1921 to 2020 with volatile year-to-year changes.

Suicide rates for US women aged above 65 gradually declined from 1921 to 2020. In Australia, the 65+ female suicide rate was overall stable, other than a peak in the 1960s (highest rate 17.7 per 100 000 in 1967).

### Sub-analysis of the 65+ age groups

**Fig. 3:**
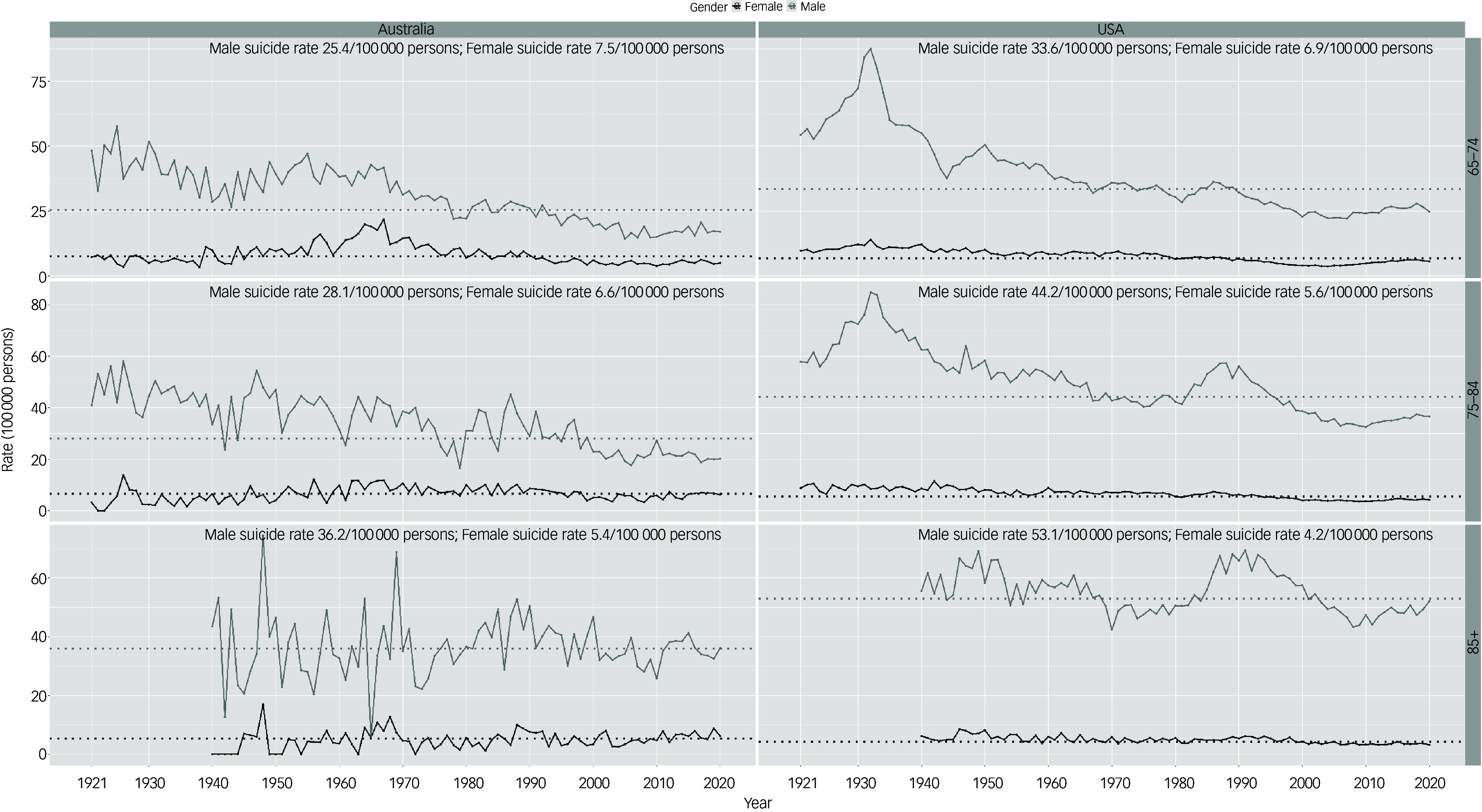
Suicide rates by age groups and gender in the USA and Australia for 65+ 10-year age groups, 1921–2020. The dotted line represents the suicide rates of males and females in Australia and the USA.

As seen in Fig. 3, men aged 65–74 in the USA had a suicide rate peak in the early 1930s (87.6 per 100 000 in 1932), while the overall rate declined from 1950 to 2020 with a minor peak in the 1980s (35.8 per 100 000 in 1986). Australian 65–74 male suicide rates overall declined from the 1920s to 2020, with volatile year-to-year changes.

Suicide rates for US women aged 65–74 remained relatively stable throughout the century, other than a minor trough in the 2000s (lowest rate 3.7 per 100 000 in 2004). The Australian 65–74 female suicide rate had a peak in the mid-1960s (highest rate 21.8 per 100 000 in 1967).

Suicide trends in US men aged 75–84 showed an overall decline from the highest rate of 84.9 per 100 000 in 1932 to the lowest rate of 32.5 per 100 000 in 2010, although there was a peak in the 1980s (57.3 per 100 000 in 1988). The Australian suicide rate in this age group had a gradual decline over the century, although there were year-to-year fluctuations.

The US suicide rate for women aged 75–84 remained relatively stable, as did that for Australia although there were greater year-to-year fluctuations.

In US men aged 85+ there was little overall change in suicide rate from 1940 to 2020, but it did display peaks in the late 1940s (69.2 per 100 000 in 1949) and early 1990s (69.5 per 100 000 in 1991). The suicide rate in Australian men aged 85+ showed greater volatility in year-to-year changes, but there was little overall change of suicide rate from 1940 to 2020.

Suicide rates in women aged 85+ in the USA appeared relatively stable. The Australian female suicide rate for age 85+ showed more volatile year-to-year changes.

## Discussion

### Male suicide trends

Male suicide rates have been the principal driver of overall suicide trends in both the USA and Australia over the past century, with the male to female rate ratio ranging from 2:1 to >5:1 for most of the period.^1,15^ Overall 100-year national suicide rates are broadly consistent with Durkheim’s observation that each country has a relatively stable suicide rate, in the absence of major events such as war, economic depression or changes in access to lethal means.^3,4^ Overall suicide rates tended to return towards the gender-specific national mean over the century. In addition, we found that the age-specific male suicide rates for youth and older adults converged towards the overall gender-specific national mean over the 20th century. While initially older male suicide rates (age 65+) were far higher than youth suicide rates (age 15–24) in 1921, older and younger males had comparable suicide rates by 2020. Overall suicide rates in the USA and Australia were similar and relatively stable from the second half of the 20th century onwards, with the exception of a peak in Australian suicide rates in the 1960s.

### Female suicide trends

Female suicide trends remained largely stable except for a marked rise in Australian women during the 1960s. The major peak of Australian female suicide rates (ages 25–44, 45–64, >65) in the 1960s was partly attributed to barbiturates being made more available through Australia’s Pharmaceutical Benefits Scheme (PBS) in 1960.^10^ In the context of regulatory changes in sedative prescriptions from July 1967, suicide rates in Australian females reduced shortly thereafter.^10^ However, US female suicide rates for women did not commensurately increase despite similar regulatory availability of barbiturates during the period of the 1950s and 1960s.^16^ US federal legislation to restrict barbiturate prescriptions was first enacted in 1951, more than a decade earlier than in Australia.^17^ Further analysis is required to understand whether the 1960s peak in Australian female suicides was evident in other countries, but it may reflect greater access to prescription medication in general made possible through Australia’s PBS.

### Youth (15–24) suicide trends

Whilst there was relative stability and similarity of overall suicide rates across the USA and Australia after 1950, there were some significant shifts within specific age groups. In particular, there were marked and ongoing increases in male youth (age 15–24) suicide rates between 1951 and 2000 and another steep increase from 2010 to 2020, which parallels global trends in adolescent mental health issues.^18–20^ This is also reflected in findings from the Australian study of mental health and well-being of 2020–2022, where Australian adolescents aged 16–24 years experienced the highest and largest increase in the rate of mental disorders compared to 2007, along with a rise in rates of suicidal ideation despite improved service use and treatment.^20,21^

Several social and cultural changes likely underpin the overall rising trend in youth suicides, including income inequality, family breakdown, youth unemployment, unstable relationships, declining religious engagement, rising imprisonment rates and substance use.^22–24^ Youth social capital has been declining since the 1960s with rising levels of individualism, materialism and neuroticism, increased vocational expectations and reduced levels of trust and loneliness.^25^ Eckersley and Dear argue, using a Durkheim framework, that the rise in youth suicides in the 20th century, ‘reflects a failure of Western societies to provide appropriate sites or sources of social identity and attachment, and, conversely, a tendency to promote unrealistic or inappropriate expectations of individual freedom and autonomy’.^26^

There have been recent concerns that the impact of social media (particularly since 2012) might have led to worsening mental health outcomes (depression and self-harm).^18^ Social media-related harm may arise from disruption of in-person social interactions, effects on sleep duration and quality, cyberbullying, toxic online interactions and contagion of online-mediated self-harm.^18^ There have been increases in youth suicide rates in both the USA and Australia in the last decade from 2011 to 2020. This impact of social media will need to be closely monitored, with possible policy implications for social media regulation.^19^

### Young adult (25–44) suicide trends

Compared to youth age groups, there were similar but less pronounced suicide rate increases for people aged 25–44 in both the USA and Australia during the latter half of the 20th century. These rates for those aged 25–44 continued to rise in the USA in the 21st century, whilst rates declined in Australia.

In Australia during the 1990s there was a major increase in premature mortality in young men because of AIDS and suicide.^27^ This was counterbalanced by a reduction of deaths in young men from other causes.^27^ HIV/AIDS emerged as a leading cause of death among young men aged 25–44 years in the USA in 1990.^28^ It is possible that the AIDS epidemic may have partially contributed to the rise in suicide in young men observed in Australia and the USA during the 1980s and 1990s. Other contributions could include the economic downturn in the early 1980s.^29^

### Middle age (45–64) suicide trends

Notably, US suicide rates for middle-aged Americans rose during the 21st century, coinciding with a major rise in opiate prescription and related opiate overdoses.^7^ This rise in US middle-aged suicide rates was not seen in Australia.^15^ This may arise from lower opiate prescription and related misuse in Australia because of greater regulatory oversight.^30^ Case and Deaton in their analysis of US mortality data in the 21st century, somewhat controversially coined the term ‘deaths of despair’ to describe the triad of deaths caused by suicide, opiates and alcohol.^7^ They speculated that the rise in middle-aged suicides was likely related to widespread opioid availability, the lack of universal healthcare coverage and widening income inequality, particularly for those without a high-school degree.^7^

### Older age (65+) suicide trends

The older age population has significantly changed from the early 20th century to the 21st century, with the result that 4.7% of the total US population were aged 65 and over in 1920 compared to 16.8% in 2020 (representing a growth rate of almost five times that of the total population). There have been similar changes in Australia, where 4.6% of the population were aged 65 and over in 1922, compared to 16% in 2020.^31,32^ The major advance in suicide reduction in Australia and the USA over the century is the progressive reduction in older male suicides. This may have been related to improvements in the recognition and care of both physical and mental disorders in the elderly, mediated through better health and welfare services.^33,34^ In general, the socioeconomic position of the elderly has also improved significantly since the 1960s, with reductions in poverty levels, related to improved social security support, availability of superannuation/ retirement funds and rising asset values (housing and shares).^24^ The above improvements may have led to increased social status of seniors and reduced feelings of redundancy.^33^

Whilst the combined trend for the older age group overall declined through the century, most of the downtrend occurred for age groups between 65 and 84, while the suicide rate in those aged 85+ remained stable. The late old age population remains at high risk as a result of biopsychosocial risk factors, including increased numbers of widowed or divorced individuals leading, in turn, to higher rates of isolation, high rates of major affective illnesses including depression and poorer physical health, leading to challenges in accessing medical care.^35^

### Overall suicide trends

Based on Durkheim’s work in the late 1800s, societal disturbances or crises that create a stronger integration of society are associated with lower suicide rates, and the inverse is true for a society that disintegrates.^4^ Consistent with this observation, troughs in suicide rates corresponded with both World War I (1914–1918) and WWII (1939–1945) in the USA and Australia.^6,15^ By contrast, the highest annual suicide rates were recorded in the USA in 1932 and Australia in 1930 during the Great Depression – a period of high unemployment and high social disintegration.^5,15^ The Australian Gun Law Reforms introduced in 1996 following a gun massacre in the country corresponded to a decline in firearm-related and overall suicides after this period.^15^ Rates of suicide related to this method declined largely from 1987, continuing post 1996.^15^

As a result of cumulative changes for younger and older males, suicide rates across the age groups were more similar than a century ago, converging towards the mean for all age groups combined. In 1921, US male suicide rates ranged from 8.3 per 100 000 population (for young males) to 55.9 (in older males), but by 2020 the discrepancy was reduced to 22.4–30.9. In Australia, the male suicide rate ranged from 8.1 per 100 000 population (for young males) to 45.6 (in older males) in 1921, but rates were approximately equal by 2020 (21.2 for younger males and 20.0 in older males). Male rates of suicide across the age groupings have moved closer to the overall national mean rates of male suicides in the USA and Australia. There is greater equivalence in suicide risk across the lifespan, but more years of life lost with youth suicide.

### Limitations and future research

Limitations of this study include the potential inaccuracy of the registration of deaths and misclassification of cause of death. Underreporting and misclassification of suicide deaths may have been more prominent, albeit to varying degrees, at the beginning of the 20th century in both the USA and Australia compared to contemporary reporting, leading to inaccuracies when comparing more recent suicide trends to those at the beginning of the study.^36^ Previous studies have demonstrated that suicides have historically been mislabelled as accidental harm when they occurred through means of substance use such as prescription opioids, and intoxication cases were more likely to be reported as an undetermined manner of death when compared to other violent methods such as gunshot or hanging.^37^ It is likely that the start of the opioid crisis in the USA contributed even more to the inaccuracy of suicide rates.^37^ In the case of Australia, changes to the data collection system from 2000 to 2006 may have led in 2000 to an underreporting of suicides until the system was further modified in 2006.^38^

Both Australia and the USA are countries that have citizens who have emigrated from a vast range of countries, faiths and sociocultural contexts, as well as indigenous populations. Indigenous populations in the USA and Australia were not included in this study as the USA only provided First Nations data from 1999 and Australia only from 2001, and these data did not include age stratification. Neither nation provides adequate data on suicides among indigenous populations over the past century, partly because individuals’ indigenous status is often unreported.^39^ Analysis by ethnicity and related factors were also not included, as the USA only provided this data from 1999 onwards, and we were not able to find Australian suicide data categorised specifically by ethnicity. It would be interesting to compare these >100-year suicide trends seen in the USA and Australia with those in similar Anglophone countries with comparable well-documented suicide records. Suicide records over 100 years only included male and female categories and there were historically no subcategories for transgender or non-binary individuals. Gender identity has been an emergent issue in both countries – this too, merits future research, predicated on the collection of such categories.

From a Durkheimian perspective, overall national suicide rates were relatively stable in the USA and Australia from 1921 to 2020, except when there were major events such as war or economic downturns. There were broad similarities in US and Australian suicide rates, particularly since 1950. In both countries male suicide rates varied more than female suicide rates during the last 100 years, with the exception of rising female suicide rates in Australia in the 1960s. However, there were major shifts in male suicide rates for various age groups. Over the century, the suicide risk for younger and older males moved towards the overall gender-specific national mean suicide rate. This meant that years-of-life-lost because of youth suicide has increased in the USA and Australia since the 1950s with the rising suicide rates amongst males aged 15–24. The major gains in the USA and Australia have been lower suicide rates amongst older males aged 65–84, while the rate in the 85+ male population remained high through the century. As a result of these cumulative changes for younger and older males, suicide rates across the age groups moved towards the century national mean for all male age groups combined. This might reflect changes in social alienation or anomie with less protection of young men and more inclusion of older males.

The socioeconomic drivers of these sustained shifts in suicide rates towards the overall national age and gender means need to be better understood. This may require further analysis of broader socioeconomic trends or events as potential determinants of long-term changes in gender- and age-specific suicide rates. Further research could segment suicide trends into smaller and more homogenous socioeconomic groups, and compare a wider range of countries to determine the relative impact of sociocultural, economic and climate changes on youth and older male suicide rates, as well as examine ethnicity and related identity factors as available from the population-level data for each country.

## Supporting information

Ma et al. supplementary material Ma et al. supplementary material

## Data Availability

The data are publicly available from the Australian Institute of Health and Welfare and Centers for Disease Control and Prevention.

## Supplementary material

The supplementary material is available online at https://doi.org/10.1192/bjo.2025.10863
