# Supplementary material for: Comparison of suicide rates in the USA and Australia between 1921 and 2020: major shifts in youth and elderly suicide rates over a century
Source: BJPsych Open. 2025 Oct 30;11(6):e253. doi: 10.1192/bjo.2025.10863 (PMC12641414; doi:10.1192/bjo.2025.10863)
Supplement: Ma et al. supplementary material [file S2056472425108636sup001.docx]

**Supplementary data:**

Table 1: Incidence rate ratio of US and Australian suicide rate eras by gender and decade (relative to baseline period 1921-1930).

| **Era** | **US** | | | **Australia** | | |
| --- | --- | --- | --- | --- | --- | --- |
|  | **IRR^$^** | **95% CI^%^** | **p-value** | **IRR^$^** | **95% CI^%^** | **p-value** |
| **Male** | | | | | | |
| **1921-1930^§^** | - | - | - | - | - | - |
| **1931-1940** | 1.15 | 1.14-1.16 | <0.001 | 0.89 | 0.86-0.92 | <0.001 |
| **1941-1950** | 0.84 | 0.84-0.85 | <0.001 | 0.63 | 0.61-0.66 | <0.001 |
| **1951-1960** | 0.83 | 0.83-0.84 | <0.001 | 0.83 | 0.81-0.86 | <0.001 |
| **1961-1970** | 0.84 | 0.84-0.85 | <0.001 | 0.95 | 0.92-0.98 | 0.004 |
| **1971-1980** | 0.88 | 0.88-0.89 | <0.001 | 0.81 | 0.79-0.84 | <0.001 |
| **1981-1990** | 0.92 | 0.91-0.93 | <0.001 | 0.92 | 0.89-0.94 | <0.001 |
| **1991-2000** | 0.87 | 0.86-0.87 | <0.001 | 1.01 | 0.98-1.04 | 0.7 |
| **2001-2010** | 0.84 | 0.83-0.85 | <0.001 | 0.80 | 0.78-0.83 | <0.001 |
| **2011-2020** | 0.97 | 0.97-0.98 | <0.001 | 0.85 | 0.83-0.88 | <0.001 |
| **Female** | | | | | | |
| **1921-1930^§^** | - | - | - | - | - | - |
| **1931-1940** | 1.07 | 1.05-1.09 | <0.001 | 1.09 | 1.01-1.17 | 0.022 |
| **1941-1950** | 0.84 | 0.83-0.85 | <0.001 | 1.01 | 0.94-1.08 | 0.9 |
| **1951-1960** | 0.73 | 0.71-0.74 | <0.001 | 1.31 | 1.23-1.40 | <0.001 |
| **1961-1970** | 0.95 | 0.93-0.96 | <0.001 | 2.10 | 1.97-2.23 | <0.001 |
| **1971-1980** | 0.96 | 0.95-0.97 | <0.001 | 1.51 | 1.42-1.61 | <0.001 |
| **1981-1990** | 0.76 | 0.75-0.77 | <0.001 | 1.13 | 1.06-1.21 | <0.001 |
| **1991-2000** | 0.62 | 0.61-0.63 | <0.001 | 1.08 | 1.02-1.15 | 0.013 |
| **2001-2010** | 0.66 | 0.65-0.67 | <0.001 | 0.97 | 0.91-1.03 | 0.3 |
| **2011-2020** | 0.83 | 0.82-0.84 | <0.001 | 1.17 | 1.11-1.25 | <0.001 |

^$^Incidence Rate Ratio, ^%^Confidence Interval, ^§^Reference group
